# Supplementary material for: Equity Analysis of Repeated Cross-Sectional Survey Data on Mental Health Outcomes in Saskatchewan, Canada during COVID-19 Pandemic
Source: Int J Environ Res Public Health. 2022 Oct 24;19(21):13808. doi: 10.3390/ijerph192113808 (PMC9655244; doi:10.3390/ijerph192113808)
Supplement: Supplementary file 1 [file ijerph-19-13808-s001.zip › ijerph-1934032-supplementary.pdf]

Table S1. Bivariate results of self-reported anxiety for each study cycle.

Variables		Study cycle 2 (August 2020)			Study wave 5 (February 2021)			Study cycle 7 (June 2021)		
		Mild	*Moderate*	*Severe*	*Mild*	*Moderate*	*Severe*	*Mild*	*Moderate*	*Severe*
**Current age (years)**										
	16 to 29	23 (25.3)	37 (40.7)	31 (34.1)	29 (28.4)	37 (36.3)	36 (35.3)	41 (36.9)	37 (33.3)	33 (29.7)
	30 to 49	60 (31.4)	81 (42.4)	50 (26.2)	59 (29.4)	78 (38.8)	64 (31.8)	74 (34.7)	92 (43.2)	47 (22.1)
	50 and above	145 (55.1)	84 (31.9)	34 (12.9)	132 (52.6)	75 (29.9)	44 (17.5)	124 (50.2)	82 (33.2)	41 (16.6)
	χ^2 (df), p	*43.60 (4),* ***<0.001***			*34.87 (4),* ***<0.001***			*17.26 (4),* ***0.002***		
**Gender**										
	Men	119 (45.4)	95 (36.3)	48 (18.3)	124 (47.0)	89 (33.7)	51 (19.3)	129 (46.7)	95 (34.4)	52 (18.8)
	Women	108 (38.4)	107 (38.1)	66 (23.5)	96 (34.0)	100 (35.5)	86 (30.5)	108 (37.0)	115 (39.4)	69 (23.6)
	χ^2 (df), p	*3.42 (2), 0.180*			*12.56 (2),* ***0.002***			*5.70 (2), 0.058*		
**Location of residence**										
	Mid-size city/town	38 (50.7)	26 (34.7)	11 (14.7)	33 (37.9)	30 (34.5)	24 (27.6)	30 (39.0)	32 (41.6)	15 (19.5)
	Rural	68 (50.0)	43 (31.6)	25 (18.4)	56 (40.6)	45 (32.6)	37 (26.8)	85 (50.6)	54 (32.1)	29 (17.3)
	Regina	55 (30.4)	81 (44.8)	45 (24.9)	59 (36.9)	56 (35.0)	45 (28.1)	64 (37.6)	58 (34.1)	48 (28.2)
	Saskatoon	68 (44.2)	53 (34.4)	33 (21.4)	72 (42.6)	58 (34.3)	39 (23.1)	60 (38.7)	66 (42.6)	29 (18.7)
	χ^2 (df), p	*16.96 (6),* ***0.009***			*1.85 (6), 0.933*			*13.24 (6),* ***0.039***		
**Household composition**										
	Live alone	53 (41.7)	43 (33.9)	31 (24.4)	44 (38.3)	35 (30.4)	36 (31.3)	51 (44.0)	40 (34.5)	25 (21.6)
	Live with others	176 (42.0)	159 (37.9)	84 (20.0)	176 (40.1)	155 (35.3)	108 (24.6)	188 (41.3)	171 (37.6)	96 (21.1)
	χ^2 (df), p	*1.32 (2), 0.516*			*2.28 (2), 0.319*			*0.40 (2), 0.817*		
**Parental status**										
	Not a parent	153 (46.5)	111 (33.7)	65 (19.8)	140 (40.5)	107 (30.9)	99 (28.6)	137 (41.8)	115 935.1)	76 (23.2)
	Has children ≤ 17y	39 (30.0)	56 (43.1)	35 (26.9)	48 (37.5)	51 (39.8)	29 (22.7)	65 (45.5)	55 (38.5)	23 (16.1)
	Has children ≥ 18y	29 (42.0)	28 (40.6)	12 (17.4)	27 (41.5)	24 (36.9)	14 (21.5)	32 (36.4)	34 (38.6)	22 (25.0)
	Has children in both age groups	8 (47.1)	7 (41.2)	2 (11.8)	5 (35.7)	7 (50.0)	2 (14.3)	4 (33.3)	7 (58.3)	1 (8.3)

χ² (df), p	*12.24 (6), 0.057*			*6.45 (6), 0.375*			*7.11 (6), 0.311*		
**Employment status**									
Unemployed	16 (25.8)	27 (43.5)	19 (30.6)	14 (22.6)	16 (25.8)	32 (51.6)	9 (17.6)	25 (49.0)	17 (33.3)
Retired/Student	81 (56.3)	44 (30.6)	19 (13.2)	80 (50.3)	49 (30.8)	30 (18.9)	94 (58.8)	35 (21.9)	31 (19.4)
Employed	131 (39.8)	128 (38.9)	70 (21.3)	125 (37.8)	123 (37.2)	83 (25.1)	134 (38.1)	149 (42.3)	69 (19.6)
χ² (df), p	*20.67 (4),* ***<0.001***			*30.72 (4),* ***<0.001***			*37.93 (4),* ***<0.001***		
**Highest level of education**									
Elementary/high school	66 (43.1)	52 (34.0)	35 (22.9)	66 (42.6)	43 (27.7)	46 (29.7)	87 (49.2)	52 (29.4)	38 (21.5)
College/technical	74 (40.7)	71 (39.0)	37 (20.3)	62 (35.6)	65 (37.4)	47 (27.0)	62 (37.8)	66 (40.2)	36 (22.0)
University	82 (40.2)	80 (39.2)	42 (20.6)	91 (41.7)	77 (35.3)	50 (22.9)	89 (39.2)	91 (40.1)	47 (20.7)
χ² (df), p	*1.27 (4), 0.866*			*5.38 (4), 0.250*			*7.18 (4), 0.126*		
**Income**									
< $20K	9 (30.0)	13 (43.3)	8 (26.7)	11 (28.2)	10 (25.6)	18 (46.2)	4 (19.0)	7 (33.3)	10 (47.6)
$20K to $49K	45 (39.1)	40 (34.8)	30 (26.1)	37 (27.4)	47 (34.8)	51 (37.8)	33 (29.5)	44 (39.3)	35 (31.3)
$50K to $99K	92 (42.8)	83 (38.6)	40 (18.6)	80 (42.8)	69 (36.9)	38 (20.3)	118 (49.4)	82 (34.3)	39 (16.3)
≥ $100K	64 (47.8)	52 (38.8)	18 (13.4)	77 (50.0)	52 (33.8)	25 (16.2)	64 (41.3)	61 (39.4)	30 (19.4)
χ² (df), p	*8.85 (6), 0.182*			*33.73 (6),* ***<0.001***			*25.78 (6),* ***<0.001***		
**Immigration status**									
Born outside Canada	59 (42.1)	48 (34.3)	33 (23.6)	53 (39.8)	44 (33.1)	36 (27.1)	56 (42.1)	41 (30.8)	36 (27.1)
Born in Canada	166 (41.8)	150 (37.8)	81 (20.4)	165 (41.3)	137 (34.3)	98 (24.5)	178 (41.6)	167 (39.0)	83 (19.4)
χ² (df), p	*1.41 (4), 0.841*			*9.74 (4),* ***0.045***			*4.97 (4), 0.290*		
**Ethnicity**									
Indigenous/ Visible minority (African, Asian, Hispanic/Latino/Others)		Not available		29 (43.9)	22 (33.3)	15 (22.7)	47 (47.0)	34 (34.0)	19 (19.0)
White				191 (39.1)	168 (34.4)	130 (26.6)	192 (40.9)	176 (37.4)	102 (21.7)
χ² (df), p				*0.69 (2), 0.705*			*1.29 (2), 0.524*		

**Physical disability**										
	Yes		Not available		7 (24.1)	12 (41.4)	10 (34.5)	9 (26.5)	10 (29.4)	15 (44.1)
	No				212 (40.5)	177 (33.8)	134 (25.6)	229 (42.8)	200 (37.4)	106 (19.8)
	χ^2 *(df), p*				*3.14 (2), 0.208*			*11.47 (2),* ***0.003***		

Table S2. Bivariate results of self-reported depression for each study cycle.

Variables	Study cycle 2 (August 2020)			Study cycle 5 (February 2021)			Study cycle (June 2021)		
	Low	*Moderate*	*High*	*Low*	*Moderate*	*High*	*Low*	*Moderate*	*High*
**Current age (years)**									
16 to 29	31 (33.7)	38 (41.3)	23 (25.0)	32 (33.7)	34 (35.8)	29 (30.5)	51 (47.2)	35 (32.4)	22 (20.4)
30 to 49	89 (47.3)	61 (32.4)	38 (20.2)	91 (45.0)	65 (32.2)	46 (22.8)	103 (49.3)	78 (37.3)	28 (13.4)
50 and above	183 (69.8)	53 (20.2)	26 (9.9)	169 (67.3)	53 (21.1)	29 (11.6)	175 (71.1)	49 (19.9)	22 (8.9)
χ^2 *(df), p*	*45.21 (4),* ***<0.001***			*41.83 (4),* ***<0.001***			*32.37 (4),* ***<0.001***		
**Gender**									
Men	148 (56.7)	64 (24.5)	49 (18.8)	149 (56.7)	67 (25.5)	47 (17.9)	172 (63.2)	69 (25.4)	31 (11.4)
Women	153 (54.8)	88 (31.5)	38 (13.6)	143 (51.3)	84 (30.1)	52 (18.6)	156 (54.5)	91 (31.8)	39 (13.6)
χ^2 *(df), p*	*4.66 (2), 0.097*			*1.81 (2), 0.403*			*4.37 (2), 0.112*		
**Location of residence**									
Mid-size city/town	47 (61.0)	20 (26.0)	10 (13.0)	44 (51.2)	23 (26.7)	19 (22.1)	44 (57.9)	24 (31.6)	8 (10.5)
Rural	83 (61.5)	31 (23.0)	21 (15.6)	78 (56.1)	37 (26.6)	24 (17.3)	107 (64.8)	44 (26.7)	14 (8.5)
Regina	88 (50.0)	56 (31.8)	32 (18.2)	74 (46.8)	51 (32.3)	33 (20.9)	100 (59.5)	36 (21.4)	32 (19.0)
Saskatoon	85 (55.2)	45 (29.2)	24 (15.6)	96 (57.8)	42 (25.3)	28 (16.9)	77 (50.7)	58 (38.2)	17 (11.2)
χ^2 *(df), p*	*5.54 (6), 0.476*			*5.12 (6), 0.528*			*19.22 (6),* ***0.004***		
**Household composition**									
Live alone	69 (53.9)	32 (25.0)	27 (21.1)	53 (45.7)	32 (27.6)	31 (26.7)	61 (53.5)	31 (27.2)	22 (19.3)
Live with others	234 (56.5)	120 (29.0)	60 (14.5)	240 (55.3)	121 (27.9)	73 (16.8)	267 (59.7)	131 (29.3)	49 (11.0)
χ^2 *(df), p*	*3.32 (2), 0.189*			*6.33 (2),* ***0.042***			*5.73 (2), 0.057*		
**Parental status**									
Not a parent	186 (57.1)	89 (27.3)	51 (15.6)	170 (50.1)	95 (28.0)	74 (21.8)	180 (55.9)	95 (29.5)	47 (14.6)
Has children ≤ 17y	62 (47.7)	43 (33.1)	25 (19.2)	70 (54.7)	38 (29.7)	20 (15.6)	88 (63.3)	38 (27.3)	13 (9.4)
Has children ≥ 18y	42 (60.0)	17 (24.3)	11 (15.7)	42 (61.8)	19 (27.9)	7 (10.3)	55 (61.8)	24 (27.0)	10 (11.2)
Has children in both age groups	14 (77.8)	3 (16.7)	1 (5.6)	9 (64.3)	2 (14.3)	3 (21.4)	6 (50.0)	5 (41.7)	1 (8.3)

	χ² (df), p	*7.89 (6), 0.246*			*7.94 (6), 0.242*			*4.66 (6), 0.587*		
**Employment status**										
	Unemployed	27 (42.9)	23 (36.5)	13 (20.6)	23 (37.1)	18 (29.0)	21 (33.9)	20 (39.2)	25 (49.0)	6 (11.8)
	Retired/Student	103 (72.0)	29 (20.3)	11 (7.7)	103 (66.9)	33 (21.4)	18 (11.7)	115 (72.8)	24 (15.2)	19 (12.0)
	Employed	172 (52.9)	95 (29.2)	58 (17.8)	164 (49.7)	101 (30.6)	65 (19.7)	190 (55.1)	112 (32.5)	43 (12.5)
	χ² (df), p	*21.60 (4),* ***<0.001***			*24.03 (4),* ***<0.001***			*28.27 (4),* ***<0.001***		
**Highest level of education**										
	Elementary/high	80 (52.3)	46 (30.1)	27 (17.6)	77 (48.7)	48 (30.4)	33 (20.9)	101 (57.7)	49 (28.0)	25 (14.3)
	College/technical	102 (56.0)	52 (28.6)	28 (15.4)	87 (50.3)	51 (29.5)	35 (20.2)	90 (56.3)	52 (32.5)	18 (11.3)
	University	116 (57.7)	54 (26.9)	31 (15.4)	127 (58.8)	54 (25.0)	35 (16.2)	136 (61.3)	59 (26.6)	27 (12.2)
	χ² (df), p	*1.13 (4), 0.889*			*4.61 (4), 0.329*			*2.28 (4), 0.684*		
**Income**										
	< $20K	12 (37.5)	13 (40.6)	7 (21.9)	13 (33.3)	15 (38.5)	11 (28.2)	5 (26.3)	11 (57.9)	3 (15.8)
	$20K to $49K	62 (53.0)	36 (30.8)	19 (16.2)	51 (37.8)	42 (31.1)	42 (31.1)	53 (46.9)	40 (35.4)	20 (17.7)
	$50K to $99K	125 (58.7)	54 (25.4)	34 (16.0)	106 (58.2)	45 (24.7)	31 (17.0)	155 (65.7)	57 (24.2)	24 (10.2)
	≥ $100K	78 (59.1)	39 (29.5)	15 (11.4)	102 (65.4)	42 (26.9)	12 (7.7)	99 (64.7)	38 (24.8)	16 (10.5)
	χ² (df), p	*7.64 (6), 0.265*			*40.22 (6),* ***<0.001***			*22.94 (6),* ***0.001***		
**Immigration status**										
	Born outside Canada	75 (54.0)	40 (28.8)	24 (17.3)	73 (57.5)	29 (22.8)	25 (19.7)	72 (55.4)	39 (30.0)	19 (14.6)
	Born inside Canada	224 (57.1)	108 (27.6)	60 (15.3)	218 (54.2)	115 (28.6)	69 (17.2)	251 (59.5)	121 (28.7)	50 (11.8)
	χ² (df), p	*1.57 (4), 0.813*			*20.80 (4),* ***<0.001***			*1.66 (4), 0.797*		
**Ethnicity**										
	Indigenous/ Visible minority (African, Asian, Hispanic/Latino/Others)		Not available		32 (50.0)	16 (25.0)	16 (25.0)	58 (59.8)	23 (23.7)	16 (16.5)
	White				260 (53.6)	137 (28.2)	88 (18.1)	270 (58.3)	139 (30.0)	54 (11.7)
	χ² (df), p				*1.75 (2), 0.416*			*2.63 (2), 0.268*		
**Physical disability**										

	Yes		Not available		12 (40.0)	8 (26.7)	10 (33.3)	17 (48.6)	15 (42.9)	3 (8.6)
	No				280 (54.1)	145 (28.0)	93 (18.0)	311 (59.1)	147 (27.9)	68 (12.9)
	χ^2 (df), p				*4.63 (2), 0.098*			*3.64 (2), 0.162*		

Table S3. Bivariate results of accessing mental health support and services for each study cycle.

**Variables**		**Study cycle 2 (August 2020)**			**Study cycle 5 (February 2021)**			**Study cycle 7 (June 2021)**		
		Needed support and received	*Needed but no access*	*Did not need and did not access*	*Needed support and received*	*Needed but no access*	*Did not need and did not access*	*Needed support and received*	*Needed but no access*	*Did not need and did not access*
**Current age (years)**										
	16 to 29	31 (33.0)	16 (17.0)	47 (50.0)	44 (44.0)	12 (12.0)	44 (44.0)	33 (28.7)	18 (15.7)	64 (55.7)
	30 to 49	52 (27.1)	22 (11.5)	118 (61.5)	65 (31.7)	27 (13.2)	113 (55.1)	57 (26.5)	29 (13.5)	129 (60.0)
	50 and above	21 (8.0)	20 (7.6)	222 (84.4)	25 (9.8)	25 (9.8)	206 (80.5)	24 (9.6)	23 (9.2)	204 (81.3)
	χ^2 *(df), p*	*54.24 (4),* ***<0.001***			*64.11 (4),* ***<0.001***			*37.36 (4),* ***<0.001***		
**Gender**										
	Men	57 (21.6)	20 (7.6)	187 (70.8)	55 (20.6)	25 (9.4)	187 (70.0)	42 (14.9)	23 (8.2)	217 (77.0)
	Women	46 (16.4)	36 (12.8)	199 (70.8)	73 (25.5)	37 (12.9)	176 (61.5)	69 (23.5)	46 (15.6)	179 (60.9)
	χ^2 *(df), p*	*5.59 (2), 0.061*			*4.54 (2), 0.103*			*17.63 (2),* ***<0.001***		
**Location of residence**										
	Mid-size city/town	10 (13.2)	10 (13.2)	56 (73.7)	22 (25.6)	8 (9.3)	56 (65.1)	11 (14.5)	14 (18.4)	51 (67.1)
	Rural	21 (15.3)	14 (10.2)	102 (74.5)	25 (17.7)	15 (10.6)	101 (71.6)	25 (14.6)	18 (10.5)	128 (74.9)
	Regina	43 (23.6)	20 (11.0)	119 (65.4)	46 (28.6)	20 (12.4)	95 (59.0)	38 (22.2)	16 (9.4)	117 (68.4)
	Saskatoon	28 (18.4)	14 (9.2)	110 (72.4)	40 (23.4)	20 (11.7)	111 (64.9)	40 (24.7)	21 (13.0)	101 (62.3)
	χ^2 *(df), p*	*6.40 (6), 0.379*			*6.25 (6), 0.395*			*11.90 (6), 0.064*		
**Household composition**										
	Live alone	31 (24.0)	15 (11.6)	83 (64.3)	40 (34.5)	9 (7.8)	67 (57.8)	27 (22.7)	16 (13.4)	76 (63.9)
	Live with others	72 (17.2)	42 (10.0)	304 (72.7)	94 (21.1)	55 (12.4)	296 (66.5)	86 (18.7)	54 (11.7)	321 (69.6)
	χ^2 *(df), p*	*3.64 (2), 0.162*			*9.67 (2),* ***0.008***			*1.48 (2), 0.476*		
**Parental status**										
	Not a parent	55 (16.5)	37 (11.1)	240 (72.3)	85 (24.4)	36 (10.3)	227 (65.2)	67 (19.8)	46 (13.6)	225 (66.6)
	Has children ≤ 17y	38 (29.7)	15 (11.7)	75 (58.6)	35 (26.7)	18 (13.7)	78 (59.5)	23 (16.2)	16 (11.3)	103 (72.5)

	Has children ≥ 18y	6 (8.8)	4 (5.9)	58 (85.3)	12 (17.1)	8 (11.4)	50 (71.4)	20 (23.0)	6 (6.9)	61 (70.1)
	Has children in both age groups	3 (16.7)	2 (11.1)	13 (72.2)	3 (21.4)	2 (14.3)	9 (64.3)	3 (25.0)	1 (8.3)	8 (66.7)
	χ^2 (df), p	*19.17 (6),* ***0.004***			*3.96 (6), 0.682*			*4.93 (6), 0.553*		
**Employment status**										
	Unemployed	15 (24.6)	10 (16.4)	36 (59.0)	24 (39.3)	11 (18.0)	26 (42.6)	9 (18.4)	12 (24.5)	28 (57.1)
	Retired/Student	9 (6.1)	7 (4.7)	132 (89.2)	20 (12.2)	16 (9.8)	128 (78.0)	24 (14.2)	16 (9.5)	129 (76.3)
	Employed	74 (22.6)	38 (11.6)	215 (65.7)	90 (27.0)	37 (11.1)	206 (61.9)	78 (22.1)	38 (10.8)	237 (67.1)
	χ^2 (df), p	*33.32 (4),* ***<0.001***			*29.06 (4),* ***<0.001***			*14.10 (4),* ***0.007***		
**Highest level of education**										
	Elementary/high school	16 (10.1)	16 (10.1)	127 (79.9)	38 (23.0)	22 (13.3)	105 (63.6)	30 (16.6)	26 (14.4)	125 (69.1)
	College/technical	38 (21.3)	15 (8.4)	125 (70.2)	42 (24.7)	23 (13.5)	105 (61.8)	28 (16.6)	21 (12.4)	120 (71.0)
	University	49 (23.9)	26 (12.7)	130 (63.4)	54 (24.3)	20 (9.0)	148 (66.7)	56 (24.9)	22 (9.8)	147 (65.3)
	χ^2 (df), p	*14.92 (4),* ***0.005***			*2.72 (4), 0.605*			*7.06 (4), 0.133*		
**Income**										
	< $20K	9 (26.5)	7 (20.6)	18 (52.9)	20 (51.3)	5 (12.8)	14 (35.9)	6 (30.0)	8 (40.0)	6 (30.0)
	$20K to $49K	25 (21.4)	13 (11.1)	79 (67.5)	43 (31.4)	20 (14.6)	74 (54.0)	20 (17.2)	20 (17.2)	76 (65.6)
	$50K to $99K	42 (19.4)	22 (10.2)	152 (70.4)	40 (21.4)	18 (9.6)	129 (69.0)	54 (21.7)	16 (6.4)	179 (71.9)
	≥ $100K	20 (14.8)	11 (8.1)	104 (77.0)	26 (16.6)	15 (9.6)	116 (73.9)	26 (16.8)	18 (11.6)	111 (71.6)
	χ^2 (df), p	*9.09 (6), 0.168*			*30.96 (6),* ***<0.001***			*30.75 (6),* ***<0.001***		
**Immigration status**										
	Born outside Canada	32 (22.9)	8 (5.7)	100 (71.4)	28 (20.9)	5 (3.7)	101 (75.4)	25 (18.5)	11 (8.1)	99 (73.3)
	Born inside Canada	71 (17.6)	48 (11.9)	284 (70.5)	98 (24.1)	53 (13.0)	256 (62.9)	88 (20.1)	59 (13.5)	291 (66.4)
	χ^2 (df), p	*6.94 (4), 0.139*			*24.64 (4),* ***<0.001***			*6.06 (4), 0.194*		
**Ethnicity**										
	Indigenous/ Visible minority (African,		Not available		20 (30.8)	6 (9.2)	39 (60.0)	25 (24.5)	14 (13.7)	63 (61.8)

	Asian, Hispanic/Latino/Others)									
	White				114 (23.0)	58 (11.7)	324 (65.3)	88 (18.4)	56 (11.7)	334 (69.9)
	χ^2 (df), p				*2.01 (2), 0.365*			*2.69 (2), 0.260*		
**Physical disability**										
	Yes		Not available		8 (25.0)	6 (18.8)	18 (56.3)	8 (21.6)	5 (13.5)	24 (64.9)
	No				126 (23.9)	58 (11.0)	344 (65.2)	106 (19.5)	64 (11.8)	373 (68.7)
	χ^2 (df), p				*1.97 (2), 0.372*			*0.23 (2), 0.887*		

Table S4. Full regression model for factors associated with moderate, and severe anxiety compared to mild anxiety.

	**Moderate anxiety**		**Severe anxiety**	
	RRR (95% CI)	*p-value*	RRR (95% CI)	*p-value*
**Age (years)**				
16 – 29	1.47 (0.60 – 3.57)	0.388	1.97 (0.69 – 5.61)	0.200
30 – 49	2.67 (1.41 – 5.05)	**0.002**	3.47 (1.62 – 7.44)	**0.001**
Above 50	Ref.		Ref.	
**Gender**				
Women	1.73 (1.01 – 2.94)	**0.043**	2.31 (1.21 – 4.39)	**0.010**
Men	Ref.		Ref.	
**Employment status**				
Unemployed	0.99 (0.38 – 2.56)	0.989	2.10 (0.79 – 5.59)	0.136

Retired/ Student	0.74 (0.38 – 1.44)	0.380	0.67 (0.28 – 1.56)	0.360
Employed	Ref.		Ref.	
**Income**				
< $20K	0.40 (0.11 – 1.46)	0.168	2.44 (0.78 – 7.63)	0.122
$20K to $49K	1.73 (0.83 – 3.62)	0.142	3.81 (1.52 – 9.50)	**0.004**
$50K to $99K	1.26 (0.68 – 2.35)	0.451	1.24 (0.53 – 2.85)	0.611
≥ $100K	Ref.		Ref.	
**Educational status**				
Elementary/high	0.47 (0.24 – 0.93)	**0.032**	1.01 (0.45 – 2.25)	0.977
College/technical	1.10 (0.61 – 1.99)	0.729	1.83 (0.85 – 3.93)	0.116
University	Ref.		Ref.	
**Physical disability**				
Yes	2.06 (0.70 – 6.05)	0.188	1.44 (0.46 – 4.49)	0.525
No	Ref.		Ref.	
**Immigration status**				
Born outside Canada	5.24 (0.21 – 130.87)	0.313	3.83 (0.14 – 99.83)	0.419
Born inside Canada	Ref.		Ref.	
**Age × Immigration status**				
16 – 29 × born outside Canada	3.02 (0.28 – 31.70)	0.357	2.99 (0.35 – 25.68)	0.316
30 – 49 × born outside Canada	1.06 (0.18 – 6.16)	0.940	0.45 (0.11 – 1.88)	0.279
**Gender × immigration status**				
Women × born outside	0.64 (0.21 – 1.89)	0.420	0.42 (0.12 – 1.46)	0.175
**Employment × immigration status**				
Unemployed × born outside Canada	0.57 (0.08 – 4.05)	0.582	0.40 (0.05 – 2.80)	0.361
Retired/student × born outside Canada	2.18 (0.41 – 11.57)	0.357	0.51 (0.08 – 3.26)	0.484
**Income × immigration status**				

<$20K × born outside Canada	29.24 (1.42 – 601.41)	**0.029**	2.34 (0.09 – 56.48)	0.600
$20K to $49 × born outside Canada	3.17 (0.55 – 18.03)	0.192	0.73 (0.10 – 5.04)	0.753
$50K to $99K × born outside Canada	1.78 (0.37 – 8.45)	0.467	0.97 (0.18 – 5.16)	0.979
**Education × immigration status**				
Elementary/high × born outside Canada	2.06 (0.47 – 9.04)	0.337	1.36 (0.29 – 6.21)	0.687
College/technical × born outside Canada	0.78 (0.22 – 2.81)	0.712	0.19 (0.03 – 0.95)	**0.044**

Table S5. Full regression model for factors associated with moderate, and severe depression compared to mild depression.

	Moderate depression		Severe depression	
	RRR (95% CI)	*p-value*	RRR (95% CI)	*p-value*
**Age (years)**				
16 – 29	3.69 (1.48 – 9.20)	**0.005**	4.38 (1.31 – 14.66)	**0.016**
30 – 49	3.79 (1.78 – 8.09)	**0.001**	6.79 (2.72 – 16.89)	**<0.001**
Above 50	Ref.		Ref.	
**Household composition**				
Live alone	0.86 (0.43 – 1.70)	0.673	1.26 (0.57 – 2.80)	0.557
Live with others	Ref.		Ref.	
**Parental status**				
No children	3.16 (0.62 – 16.04)	0.165	0.84 (0.19 – 3.56)	0.818
Has children ≤17yrs	1.36 (0.26 – 7.03)	0.707	0.29 (0.06 – 1.29)	0.106
Has children ≥18yrs	3.90 (0.69 – 22.05)	0.123	0.93 (0.16 – 5.20)	0.938
Has children in both age group	Ref.		Ref.	
**Employment status**				
Unemployed	0.76 (0.34 – 1.70)	0.516	1.07 (0.46 – 2.50)	0.860
Retired/ Student	0.67 (0.34 – 1.31)	0.247	0.38 (0.15 – 0.96)	**0.041**
Employed	Ref.		Ref.	
**Income**				
< $20K	1.51 (0.40 – 5.60)	0.538	7.09 (1.75 – 28.72)	**0.006**
$20K to $49K	3.20 (1.47 – 6.97)	**0.003**	9.82 (3.24 – 29.71)	**<0.001**
$50K to $99K	1.68 (0.87 – 3.24)	0.121	3.31 (1.28 – 8.54)	**0.013**
≥ $100K	Ref.		Ref.	
**Educational status**				
Elementary/high	0.75 (0.37 – 1.53)	0.441	4.04 (0.63 – 25.72)	0.138

College/technical	1.39 (0.76 – 2.54)	0.278	0.78 (0.12 – 4.86)	0.795
University	Ref.		Ref.	
**Physical disability**				
Yes	1.28 (0.43 – 3.80)	0.646	2.21 (0.63 – 7.73)	0.212
No	Ref.		Ref.	
**Immigration status**				
Born outside Canada	3.09 (0.11 – 84.83)	0.504	0.95 (0.01 – 57.92)	0.982
Born inside Canada	Ref.		Ref.	
**Ethnicity**				
Visible minority	0.75 (0.14 – 3.94)	0.737	3.59 (0.91 – 14.16)	0.068
White	Ref.		Ref.	
**Age × Immigration status**				
16 – 29 × born outside Canada	4.64 (0.08 – 261.59)	0.455	48.13 (0.83 – 2776.42)	0.061
30 – 49 × born outside Canada	1.17 (0.28 – 4.89)	0.823	0.42 (0.08 – 2.03)	0.281
**Income × immigration status**				
<$20K × born outside Canada	4.56 (0.14 – 140.29)	0.385	0.52 (0.00 – 45.38)	0.775
$20K to $49K × born outside Canada	0.36 (0.06 – 1.99)	0.244	0.15 (0.01 – 1.54)	0.113
$50K to $99K × born outside Canada	0.24 (0.05 – 1.13)	0.072	0.38 (0.04 – 3.09)	0.368
**Education × immigration status**				
Elementary/high × born outside Canada	8.38 (1.60 – 43.91)	**0.012**	4.04 (0.63 – 25.72)	0.138
College/technical × born outside Canada	0.69 (0.15 – 3.16)	0.637	0.78 (0.12 – 4.86)	0.795
**Age × ethnicity**				
16 – 29 × Visible minority	0.23 (0.00 – 7.63)	0.414	0.00 (0.00 – 0.38)	**0.015**
30 – 49 × Visible minority	1.88 (0.25 – 13.65)	0.532	0.35 (0.06 – 1.98)	0.238

Table S6. Full regression model for factors associated with mental health support seeking behavior (needed support and received, needed but no access) compared to did not need and did not access.

	**Needed support and received**		**Needed but no access**	
	RR (95% CI)	*P-value*	RR (95% CI)	*P-value*
**Age (years)**				
16 – 29	6.02 (2.40 – 15.13)	**<0.001**	2.07 (0.80 – 5.32)	0.129
30 – 49	4.21 (2.05 – 8.67)	**<0.001**	1.35 (0.61 – 2.99)	0.499
Above 50	Ref.		Ref.	
**Gender**				
Men	0.65 (0.37 – 1.13)	0.133	0.78 (0.41 – 1.47)	0.449
Women	Ref.		Ref.	
**Household composition**				
Live alone	2.25 (1.14 – 4.44)	**0.018**	0.85 (0.34 – 2.12)	0.741
Live with others	Ref.		Ref.	
**Employment status**				
Unemployed	0.86 (0.39 – 1.85)	0.702	1.83 (0.78 – 4.30)	0.164
Retired/ Student	0.42 (0.17 – 1.04)	0.062	0.67 (0.28 – 1.61)	0.382
Employed	Ref.		Ref.	
**Income**				
< $20K	3.57 (1.22 – 10.43)	**0.020**	1.05 (0.23 – 4.69)	0.947
$20K to $49K	2.75 (1.19 – 6.34)	**0.018**	2.48 (1.07 – 5.74)	**0.034**
$50K to $99K	1.45 (0.71 – 2.96)	0.304	1.15 (0.53 – 2.47)	0.719
≥ $100K	Ref.		Ref.	
**Educational status**				
Elementary/high	1.13 (0.56 – 2.30)	0.717	1.23 (0.54 – 2.79)	0.617

College/technical	1.13 (0.59 – 2.17)	0.693	1.47 (0.72 – 3.02)	0.284
University	Ref.		Ref.	
**Physical disability**				
Yes	1.00 (0.29 – 3.41)	0.989	1.26 (0.40 – 3.91)	0.681
No	Ref.		Ref.	
**Immigration status**				
Born outside Canada	0.61 (0.32 – 1.14)	0.127	0.14 (0.05 – 0.43)	**0.001**
Born inside Canada	Ref.		Ref.	
**Ethnicity**				
Visible minority	0.98 (0.41 – 2.29)	0.963	1.01 (0.32 – 3.15)	0.982
White	Ref.		Ref.	
